# Experiences of mental illness stigma, prejudice and discrimination: a review of measures

**DOI:** 10.1186/1472-6963-10-80

**Published:** 2010-03-25

**Authors:** Elaine Brohan, Mike Slade, Sarah Clement, Graham Thornicroft

**Affiliations:** 1 Health Service and Population Research Department, Institute of Psychiatry, King's College London, De Crespigny Park, London SE5 8AF, UK

## Abstract

**Background:**

There has been a substantial increase in research on mental illness related stigma over the past 10 years, with many measures in use. This study aims to review current practice in the survey measurement of mental illness stigma, prejudice and discrimination experienced by people who have personal experience of mental illness. We will identify measures used, their characteristics and psychometric properties.

**Method:**

A narrative literature review of survey measures of mental illness stigma was conducted. The databases Medline, PsychInfo and the British Nursing Index were searched for the period 1990-2009.

**Results:**

57 studies were included in the review. 14 survey measures of mental illness stigma were identified. Seven of the located measures addressed aspects of perceived stigma, 10 aspects of experienced stigma and 5 aspects of self-stigma. Of the identified studies, 79% used one of the measures of perceived stigma, 46% one of the measures of experienced stigma and 33% one of the measures of self-stigma. All measures presented some information on psychometric properties.

**Conclusions:**

The review was structured by considering perceived, experienced and self stigma as separate but related constructs. It provides a resource to aid researchers in selecting the measure of mental illness stigma which is most appropriate to their purpose.

## Background

### Defining stigma

The classic starting point for defining the stigma of mental illness is Goffman's '*an attribute that is deeply discrediting'*. The recognition of this attribute leads the stigmatised person to be *'reduced... from a whole and usual person to a tainted or discounted one' *p.3 [1]. This presents stigma as the relationship between attribute and stereotype. In Goffman's terms, attributes can be categorised in three main groups: 1) abominations of the body e.g. physical disability or visible deformity, 2) blemishes of individual character e.g. mental illness, criminal conviction or 3) tribal stigmas e.g. race, gender, age.

The work of Jones and colleagues built on these categorisations with a focus on the study of 'marked relationships' [2]. In this definition, stigma occurs when the mark links the identified person via attributional processes to undesirable characteristics which discredit him or her. They propose six dimensions of stigma:

1. Concealability: how obvious or detectable a characteristic is to others

2. Course: whether the difference is life-long or reversible over time

3. Disruptiveness: the impact of the difference on interpersonal relationships

4. Aesthetics: whether the difference elicits a reaction of disgust or is perceived as unattractive

5. Origin: the causes of the difference, particularly whether the individual is perceived as responsible for this difference

6. Peril: the degree to which the difference induces feelings of threat or danger in others

Elliott and colleagues emphasised the social interaction in stigma [3]. In their definition, stigma is a form of deviance that leads others to judge an individual as illegitimate for participation in a social interaction. This occurs because of a perception that they lack the skills or abilities to carry out such an interaction, and is also influenced by judgments about the dangerousness and unpredictability of the person. Once the person is considered illegitimate then they are beyond the rules of normal social behaviour and may be ignored or excluded by the group.

There has been a substantial increase in research on mental illness related stigma over the past 10 years [4,5]. Link and Phelan note that the stigma concept has received criticism for being too individually focused and loosely defined. In response to these criticisms, they define stigma as '*the co-occurrence of its components: labeling, stereotyping, separation, status loss, and discrimination*' in a context in which power is exercised p.363 [6]. Phelan and colleagues have recently investigated the possible intersection of conceptual models of stigma and prejudice, and concluded that the two approaches have much in common with most differences being a matter of emphasis and focus. They argue that stigma and prejudice have three functions: exploitation and domination (keeping people down); disease avoidance (keeping people away) and norm enforcement (keeping people in)[7].

Corrigan has proposed a framework in which stigma is categorised as either public stigma or self stigma. Within each of these two areas, stigma is further broken down into three elements: stereotypes, prejudice and discrimination [8]. This is revised in the definition of Thornicroft et al, 2007, in which stigma includes three elements: problems of knowledge (ignorance or misinformation), problems of attitudes (prejudice), and problems of behaviour (discrimination)[9]. Sayce advocates using a discrimination framework. Stigma is presented as an unhelpful concept which prevents focus on the unfair treatment experienced by mental health service users [10].

The aim of the review is to report on survey measures assessing aspects of mental illness stigma, prejudice and discrimination reported by people personally affected by mental illness. It will review the characteristics and psychometric properties of the included measures and provide guidance regarding measures to be used in further research in the area.

### Measuring stigma

An existing review considers the measurement of mental illness stigma from multiple perspectives including mental health service users, professional groups (e.g. mental health professionals or police), the general population, families or carers of those with a mental illness and children and adolescents [11]. The current review focuses only on measures appropriate to people with personal experience of mental illness and includes several measures which have been published since the previous review in 2004. It is timely to focus on measures of the personal stigma of mental illness as these are increasingly being used as key outcomes in anti-stigma interventions [12,13]. This review will focus on the personal stigma, prejudice and discrimination associated with mental illness. For the sake of brevity, stigma will be used as an overarching term to include elements of stigma, prejudice and discrimination. In this review, the personal stigma of mental illness is considered in three main ways: perceived stigma, experienced stigma and self-stigma. Each of these aspects is defined below:

#### 1) Perceived stigma

Van Brakel and colleagues provide a definition of perceived or felt stigma research as that in which '*people with a (potentially) stigmatized health condition are interviewed about stigma and discrimination they fear or perceive to be present in the community or society*' [14]. In the original definition, felt stigma '*refers principally to the fear of enacted stigma, but also encompasses a feeling of shame associated with [the illness]' *p.33 [15]. Felt stigma may be thought of as encompassing elements of both perceived and self stigma. For the purposes of this review, perceived stigma is consistent with the definition of Van Brakel and colleagues, and does not include feelings of shame, which are instead included under self-stigma.

LeBel, highlights that perceived stigma can include both of the following [16]:

a) what an individual thinks most people believe about the stigmatised group in general

b) how the individual thinks society views him/her personally as a member of the stigmatised group

For the purposes of this review, both of these elements are included as perceived stigma.

#### 2) Experienced stigma

Van Brakel and colleagues' definition of experienced stigma as the '*experience of actual discrimination and/or participation restrictions on the part of the person affected*' will be used in this review [14]. This is similar to Scrambler & Hopkins, (1986), concept of enacted stigma or *'instances of discrimination ...on the grounds of their perceived unacceptability or inferiority' *p.33.

#### 3) Self-stigma

Corrigan and Watson, use the term public stigma to describe the ways in which the general public stigmatise people with a mental illness [17]. They describe self-stigma as the internalisation of this public stigma. An extended definition describes it as *'the product of internalisation of shame, blame, hopelessness, guilt and fear of discrimination associated with mental illness*' [18]. It has also been defined as a process, either conscious or unconscious, wherein the person with mental illness accepts diminished expectations both for and by him or herself [19]. Van Brakel et al, 2006, describe it as '*feelings of loss of self-esteem and dignity, fear, shame, guilt, etc' *In this way, it is contains elements of felt stigma as described above [15].

If self-stigma is considered as a reaction to public stigma, then it may be appropriate to also consider measures of other reactions to public stigma under this section e.g. energisation, righteous anger or no observable response [17]. The coping literature overlaps with this to a large degree, particularly with behavioural aspects of self-stigma such as disclosure or social withdrawal (See [20] for an overview of the coping literature and the Stigma Coping Orientation Scales [21,22] for further information). For the puroposes of this review these additional measures of self-stigma were not considered. The focus was soley on those which were described as measuring personal stigma.

## Method

A narrative literature review was conducted to identify survey measures of the three stigma constructs. Searching and data extraction was conducted by EB. The databases Medline, PsychInfo and the British Nursing Index were searched for published journal articles containing the title, abstract or keyword terms ('mental AND ill*' OR 'mental AND distress') AND ('stigma*' OR 'prejudic*'OR 'discriminat*') for the period 1990-2009. After removing duplicate papers, a total of 984 articles were identified. The titles and abstracts of these papers were reviewed. Papers were included if they reported on a survey measure of perceived, experienced or self-stigma which had been used with a sample of adults with a primary diagnosis of a mental illness. Only English language papers were included. As the aim was to identify measures of mental illness stigma, inclusion was not limited based on study design as long as a survey measure of mental illness stigma was used. 48 papers met these inclusion criteria. The reference lists of these papers and a personal database of stigma papers were reviewed for further papers. One systematic review of stigma and mental health was located and the reference list was checked [23]. The reference lists of 3 review papers on stigma and mental illness were also checked [6,11,24]. This resulted in the identification of a further 27 papers.

## Results

**Figure 1:**
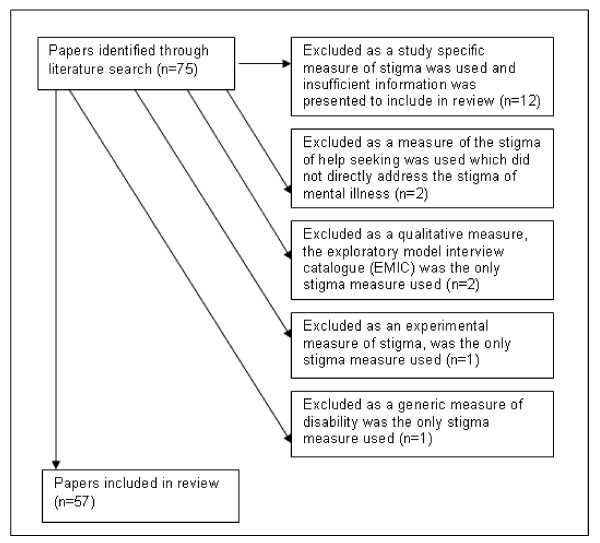
**Reasons for exclusion of papers from review**.

From the 75 identified papers, 18 were excluded. Papers were excluded for 4 main reasons: 1) a measure of stigma was created especially for the study and insufficient information was presented on the content of the measure to include 2) the paper included only a measure of a closely related constructs e.g. stigma receptivity or a generic disability scale was used as a measure of stigma and 3) the study reported only on qualitative or experimental rather than survey based measures of stigma (see figure 1).

As seen in the above figure, 57 studies were identified in the review. In these studies, 14 measures of mental illness stigma were used. Further data on all located studies is provided in Additional File 1. All but one of the papers describing the development of these measures was included in the 57 identified papers. The paper not included was published in 1987, prior to the period for this review [25].

Table 1 presents a summary of each measure. The subscales of each measure were categorised as measuring perceived, experienced or self-stigma using descriptions in the scale development papers. In cases where a subscale contained items which fell under more than one construct, the subscale was placed under the construct which represented the greatest number of items. Detail is provided on the scale structure and psychometric properties (where reported). The measures are ordered by the number of studies using the measures (final column). Table 2 presents a summary of the psychometric properties of each measure, as reported in the initial development paper. This table is a modified version of the format suggested by Terwee and colleagues for reporting on the measurement properties of health status questionnaires [26]. Terwee and colleagues highlight a framework of quality criteria for 9 aspects of psychometric assessment: content validity, internal consistency, criterion validity, construct validity, acceptability, reliability, responsiveness, floor and ceiling effects, and interpretability [26]. However, sufficient evidence was not present to use this framework consistently across the identified measures. Criterion validity or the extent to which scores relate to a gold standard was excluded due to the lack of a gold standard measure of stigma. Three additional properties based on minimal important change (acceptability, responsiveness and interpretability) were excluded as this information was not included for any located measures. Therefore Table 2 focuses on 5 properties: content validity, internal consistency, construct validity, test-retest reliability and floor or ceiling effects.

**Table 1:** Scales assessing stigma experienced by people with personal experience of mental illness

Scale	Measures Perceived stigma	Measures experienced stigma	Measures self-stigma	Measures Other	N. of studies found in review using measure
**1. Perceived Devaluation and Discrimination Scale (PDD) **[25]	Perceived discrimination (6 items) Perceived devaluation (6 items)	No	No	No	35 [22,28,33,35,44-76]
*Description*	12 item self complete measure. Each item is rated on a six-point Likert scale anchored at 1 = strongly disagree and 6 = strongly agree. The internal consistency of the scale ranges from α = 0.86 to α = 0.88 [22]				

**2. Internalised Stigma of Mental Illness (ISMI) **[33]	No	Discrimination experience (5 items)	Alienation (6 items) Stereotype endorsement (7 items) Social withdrawal (6 items)	Stigma resistance (5 items)	7 [48,69,77-81]
*Description*	29 item self complete measure. Each item is rated on a four-point Likert scale anchored at 1 = strongly disagree and 4 = strongly agree. Internal consistency (α = 0.90), test-retest reliability(*r *= 0.92)				

**3. Self-stigma of Mental Illness Scale (SSMIS) **[27]	Stereotype awareness (10 items)	No	Stereotype agreement (10 items) Stereotype self-concurrence (10 items) Self-esteem decrement (10 items)	No	5 [73,82-85]
*Description*	40 item self complete measure. Each item is rated on a 9-point Likert scale anchored at 0 = strongly disagree and 9 = strongly agree). Internal consistency for subscales range α = 0.72 to α = 0.91. Test-retest reliability for subscales ranged from 0.68-0.82. The stereotype awareness items were adapted from the PDD [25]				

**4. Consumer Experiences of Stigma Questionnaire (CESQ) **[34]	No	Experiences of stigma (9 items) Experiences of discrimination (12 items)	No	No	3 [49,86,87]
*Description*	21 item self complete postal survey. Each item is rated on a five-point Likert scale anchored at 1 = never and 5 = very often. Has also been used as an interview. Psychometric properties not reported				

**5. Rejection Experiences Scale (RES) **[47]	No	Rejection experiences (11 items)	No	No	3 [52,59,72]
*Description*	11 item self-complete scale, develsoped in Swedish. Each item rated on a 5 point Likert scale anchored 1 = never and at 5 = very often. Internal consistency α = 0.85. The scale was developed based on the 6 items from the SRES [35] and 5 items from the CESQ [34]				

**6. Depression Self-stigma Scale (DSSS)** [30]	Public stigma (4 items)	Stigmatizing experiences (6 items)	General self-stigma (9 items) Secrecy (9 items)	Treatment stigma (4 items)	1 [88]
*Description*	32 item self-complete measure. Each item rated on a 7 point Likert scale anchored at 1 = completely agree and 7 = completely disagree. Internal consistency for subscales range α = 0.78- α = 0.95 [88]				

**7. Self-reported Experiences of Rejection (SRER) **[35]	No	Rejection experiences (12 items)	No	No	1 [56]
*Description*	12 item self-complete measure. 6 items about experiences related to mental illness and 6 about experiences related to drug use. Each item is scored using a yes/no response. Internal consistency is α = 0.80. A Link and colleagues recommend the use of the CESQ rather than SRER [11]				

**8. Stigma Scale (SS)** [36]	No	Discrimination (12 items)	Disclosure (11 items)	Positive aspects (5 items)	0
*Description*	28 item self complete measure. Each item is rated on a four-point Likert scale anchored at 0 = strongly disagree and 4 = strongly agree. Test-retest reliability (kappa range 0.49-0.71) and internal consistency α = 0.87				

**9. The Inventory of Stigmatising Experiences (ISE) **[32]	Perceived stigma 2 items	Experienced stigma 2 items	Social withdrawal 1 item	Impact of stigma (5 item)	0
*Description*	10 item interview based measure with qualitative components. Each item is scored on a five point Likert Scale anchored at 1 = never and 5 = always. The scale is intended as a measure of 'the extent and impact of stigma'. Stigma experiences scale KR-20 = 0.83, stigma impact scale α = 0.91				

**10. Self-esteem and Stigma Questionnaire (SESQ) **[28]	Feelings of stigmatisation (8 items)	No	No	Self-esteem (6 items)	0
*Description*	14 item self complete measure. The feelings of stigmatisation items are adapted from the PDD (Link, 1987). It also contains 5 self-esteem items which address the respondent's confidence in their ability to complete various tasks. A sixth self-esteem item is taken from the Rosenberg self-esteem scale [89]. All items are rated on a six point Likert scale, anchored at 1 = strongly agree and 6 = strongly disagree. Internal consistency α = 0.80. Item-total correlation r = 0.4 or greater for each item. Test retest stigma scale = 0.63, self-esteem scale (0.71). α = 0.79, 0.71				

**11. Stigmatisation Scale (HSS) **[49,90]	Perceived stigma (15 items)	No	No	No	0
*Description*	15 item self-complete measure. Adapted from 18-item measure by Harvey, 2001. Each item is rated on a 5 point Likert Scale anchored at 0 = never and 4 = always. Internal consistency α ≥ 0.80				

**12. MacArthur Foundation Midlife Development in the United States (MIDUS) **[37]	No	Major discrimination (11 items) Day to day discrimination (11 items)	No	No	0
*Description*	22 item interview based measure. Each item was rated on a 5-point Likert scale anchored at 1 = all of the time and 5 = never. Assess discrimination for any reasons including disability, gender, ethnicity/race, age, religion, physical appearance, SES and other reasons. The disability category was further split into physical and mental disability. Dichotomous response for each question followed by a frequency scale anchored at 1 = often and 4 = never. Internal consistency α = 0.87				

**13. Discrimination and Stigma Scale (DISC) **[31]	Anticipated discrimination (4-items)	Experienced discrimination (32 items)	No	No	0
*Description*	36 item interview based measure. All items are rated on a 7 point Likert scale anchored at -3 = strong disadvantage and 3 = strong advantage. Psychometric properties not reported				

**14. Experiences of Discrimination Scale (EDS) **[38]	No	Has discrimination occurred (1 item) Specific settings of discrimination (8 items)	No	Stressfulness of discrimination in specific settings (8 items)	0
*Description*	Interview based measure which assesses experienced discrimination resulting from mental illness and other stigmatized identities. It asks whether discrimination has occurred, what the basis for this discrimination was, whether discrimination occurred in 8 specific settings and the level of stress associated with discrimination in each setting. Modified version of the Schedule of Racist Events Scale [91]				

**Table 2:** Assessment of measurement properties of stigma measures

Scale	Content Validity^1^	Internal Consistency^2^	Construct Validity^3^	Test-retest Reliability^4^	Floor/ ceiling effects^5^
1. PDD [25]	?	?	+	0	0

2. ISMI [33]	+	+	+	+	0

3. SSMIS [27]	+	?	+	+	0

4. CESQ [34]	+	0	0	0	-

5. RES [47]	?	?	+	0	-

6. DSSS [30]	?	+	+	0	0

7. SRE [35]	?	?	+	0	0

8. SS [36]	+	+	+	+	0

9. ISE [32]	+	?	0	0	0

10. SESQ [28]	?	+	+	?	0

11. HSS [49,90]	+	?	+	0	0

12. MIDUS [37]	?	+	+	0	-

13. DISC [31]	+	0	0	0	0

14. EDS [38]	?	0	+	0	0

+ = positive rating of property, ? = indeterminate rating of property, - = negative rating of property, 0 = no information available for property

For each property^1-5 ^a positive rating of the property was made if the below criteria were met [26]

^1^Clear description is provided of the measurement aim, the target population, the concepts that are being measured, and the item selection, target population and (investigators or experts) were involved in item selection

^2 ^Factor analysis performed on adequate sample size and Cronbach's alpha calculated per dimension and Cronbach's alpha between 0.70 and 0.95

^3^Specific hypotheses were formulated and at least 75% of results are in accordance with the hypothesis

^4^ICC or weighted Kappa ≥ 0.70

^5 ^≤ 15% of respondents achieved the highest or lowest possible scores

### Measures of perceived stigma

Seven identified measures assess aspects of perceived stigma (PDD, SSMIS, ISE, HSS, SESQ, DSSS and DISC). This is the most frequently addressed aspect of mental illness stigma with 45 (79%) of the identified studies using one of these measures. The PDD scale was most commonly used (82% of studies) [25]. Validated versions of this measure are available in German, Chinese and Swedish (See Additional File 1). It measures the individual's perception of how 'most other people' view individuals with a mental illness. Corrigan & Watson, 2002, refer to this construct as stereotype awareness. In their measure, the SSMIS, they adapt the PDD to create 10 items for inclusion as their 'stereotype awareness' subscale [27]. Similarly, the 'feelings of stigmatisation' subscale of the SSEQ is an adapted 8 item version of the PDD [28]. This construct is also known as stigma consciousness [29]. The 4 item 'public stigma' subscale of the DSSS also measures stereotype awareness [30].

As mentioned, stereotype awareness is only one aspect of perceived stigma. Several of the other identified scales instead focus on personal expectations or fears of encountering stigma i.e. a personally relevant version of stereotype awareness. This is addressed in HSS, ISE & DISC. The HSS investigates perceptions of how the person feels they have been personally viewed or treated by society. The DISC contains 4 items which address anticipated discrimination, or the expectation of being stigmatised in various aspects of life [31]. In the 2 item perceived stigma subscale of the ISE, one of the items addresses stereotype awareness while the other addresses personal fear of encountering stigma [32].

All of the measures reported on aspects of content validity. Several measures did not report on whether the target population had been involved in item selection (PDD, SESQ, DSSS) so were rated as partially fulfilling the criteria as this aspect was indeterminate. Two (DSSS, SESQ) met the full criteria for internal consistency. Four measures partially met the criteria, reporting adequate Cronbach's alpha but not reporting results of a factor analysis (PDD, SSMIS, ISE, HSS). DISC did not report on internal consistency. All measures except ISE and DISC reported adequate construct validity. Information on this property is not presented for these measures. Only the SSMIS and SESQ measured test-retest reliability, with the criteria reached in the SSMIS. The SESQ partially met the criterion (≥ 0.70) as the self-esteem subscale was slightly below the criterion at r = 0.063. Evidence on floor or ceiling effects was not located for any of the measures.

### Measures of experienced stigma

Ten of the measures in Table 1 assess aspects of experienced stigma: ISMI, CESQ, RES, DSSS, SRE, SS, ISE, MIDUS, DISC and EDS. Twenty-six (46%) of the identified studies use one of these measures. In all scales, experienced stigma refers to either experiencing stigma in general or a report of experiences of stigma in specific areas of life.

The 'discrimination experience' subscale of the ISMI contains 5 items which address both perceived and general experiences of discrimination [33]. This subscale was included under the category of experienced stigma as a greater number of the scale items address this construct. The CESQ 'discrimination' subscale asks about experiences of stigma in specific areas of life [34]. In Table 1, the CESQ 'stigma' subscale is also placed under the experienced stigma construct. This decision was taken as the majority of items refer to general stigma experiences. The RES is based on 6 items from the SRES [35] and 5 items from the CESQ [34]. The SRES was developed prior to the CESQ and the developers now recommend the use of the CESQ rather than the SRES [11]

The 12 item 'discrimination' subscale of the SS asks about general stigma experiences e.g. 'have you been talked down to' and specific experiences e.g. in education [36]. Several items also address feelings about stigma. The ISE asks two general questions about experiences of stigma [32]. The DSSS 'stigma experiences' subscale contains 6 items which consider times in which the respondent may have felt stigmatised because of experiencing or disclosing depression [30]. The DISC contains 32 items which address experiences of stigma in various areas of life including work, family and mental health service use [31].

Two of the identified measures (MIDUS, EDS) examined experienced stigma as well as multiple reasons for this stigma. Both ask about the perceived reason for poor treatment including characteristics such as mental illness, disability, gender, ethnicity/race, age, religion, physical appearance, socio-economic status and other reasons. The MIDUS contains 11 items which measure 'major discrimination' and 11 items which measure 'day to day' experiences of discrimination [37]. The EDS has 8 items which address specific areas in which stigma has been experienced [38].

All of the measures reported on aspects of content validity. Four did not report on target population involvement in item selection (RSE, DSSS, SRE, EDS). Four (ISMI, DSSS, SS and MIDUS) met the full criteria for internal consistency. Three measures partially met the criteria, reporting adequate Cronbach's alpha but did not conduct a factor analysis (RES, SRE, ISE). CESQ, DISC and EDS did not report on internal consistency. All measures except CESQ, ISE, DISC reported on construct validity with adequate results. Only the ISMI and SS measured test-retest reliability, with both reaching the criterion level. Evidence on acceptable floor and ceiling effects were not available for any measures. Of those presenting information on this property (CESQ, RES and MIDUS) several items were seen to violate the criterion, receiving more than 15% of responses. Evidence on this property was not provided for other measures.

### Measures of self-stigma

Five of the measures assessed aspects of self-stigma: ISMI, SSMIS, DSSS, SS and ISE. Nineteen (33%) of the studies used one of these measures. Self-stigma contains cognitive, affective and behavioural responses to perceived or experienced stigma. All three elements were reflected in the measures located.

Three subscales of the ISMI particularly addressed self-stigma: alienation, stereotype endorsement and social withdrawal [33]. These can be considered affective, cognitive and behavioural dimensions respectively. The discrimination experience subscale was excluded as it was considered to measure experienced stigma. The stigma resistance subscale was also excluded. Three subscales of the SSMIS measure self-stigma: stereotype agreement, stereotype self-concurrence and self-esteem decrement [27]. The SS contains a 'disclosure' subscale which focuses on cognitive, affective and behavioural aspects of disclosure [36]. The ISE contains 1 item on social withdrawal [32]. Two subscales of the DSSS address self-stigma: general self-stigma and secrecy [30]. General self-stigma includes aspects of personally relevant stereotype awareness (as discussed under perceived stigma). Secrecy addresses a similar construct to the disclosure subscale of the SS, and the social withdrawal subscale of the ISMI.

All of the measures reported on content validity. The DSSS did not report on target population involvement in item selection. Three scales (ISMI, DSSS, SS) met the full criteria for internal consistency. The SSMIS and ISE partially met the criteria, reporting adequate Cronbach's alpha but not conducting a factor analysis. All measures except ISE reported on construct validity to an adequate level. The ISMI, SSMIS and SS measured test-retest reliability, with all reaching the criterion level. Evidence on acceptable floor and ceiling effects were not available for any measures.

### Other subscales

Several other subscales were identified in the review including 'stigma resistance' in the ISMI [33], 'positive aspects' in the SS [36], 'impact of stigma' in the ISE, [32], 'self-esteem' in the SESQ [28], 'treatment stigma' in the DSSS [30] and 'stressfulness of stigma events' in the EDS [38]. These subscales did not clearly fit into one of the three stigma constructs. Stigma resistance, positive aspects and self-esteem would most closely fit with self-stigma. Treatment stigma is measuring a related construct, rather than mental illness stigma. Two other measures of help-seeking, the stigma scale for receiving psychological help for depression (SSRPH) [39] and self-stigma of seeking help (SSOSH) [40], were excluded from this review for this reason (see Figure 1). Stressfulness is examining the magnitude of experienced discrimination so would most clearly fit with this subscale. These subscales highlight additional elements of stigma, not covered by the perceived, experienced and self-stigma categories, which may be useful to consider.

## Discussion

This paper examined definitions of stigma, prejudice and discrimination and presented a review of the survey measurement of mental illness stigma. Stigma was used as an over-arching term to incorporate stigma, prejudice and discrimination. The review identified 14 scales which assessed aspects of perceived, experienced and self-stigma in 57 studies. Perceived stigma was most frequently assessed in 79% of studies, followed by experienced stigma in 46% of studies and self-stigma in 33% of studies. This is in keeping with a previous review which considered the measurement of mental illness stigma among those with personal experience of mental illness [11]. It found that 50% (n = 12) of studies used a survey based measure of status loss/discrimination (expectations), 33% (n = 8) used a survey based measure of status loss/discrimination (experiences) and 13% (n = 3) measured emotional reactions. These categories broadly map on to the perceived, experienced and self-stigma categories used in this review. Although interesting to see that the ranking of areas of emphasis is the same, this should be interpreted with caution due to the different categorisations used and as the sample includes experimental and qualitative studies as well as those using survey measures this underemphasises the proportions for survey based measures alone (as used in this study).

Psychometric properties were presented in this review using an adapted version of the framework of Terwee and colleagues [26]. No measure provides acceptable evidence on all 5 properties. A variety of properties are presented for each measure and judgments about the most appropriate measure can be based considering these properties as well as the study needs. This table should be interpreted cautiously as reported properties are based on those provided in the initial development paper and those which were not identified may be published elsewhere. Several measures including the CESQ, ISE, DISC and EDS provided information on a limited number of the measurement properties. These measures cannot be recommended for use without further work to establish these properties. Also, if not already established (see Additional File 1) further validation is necessary for all measures when used in clinical or cultural contexts which are different from the original purpose.

## Conclusions

The paper has provided an overview of commonly used measures of personal mental illness stigma, as a resource to provide guidance on which measure may be most appropriate in future research. This contributes evidence to support the evaluation of outcomes as part of anti-stigma campaigns or social inclusion interventions, fitting with the Medical Research Council's guidance on developing and evaluating complex interventions [41]. It builds on existing reviews by exploring this area of stigma measurement in detail and including recently developed measures.

This review has focused on survey measures. However, as mentioned in the discussion alternative methods of considering this topic such as qualitative and experimental investigations e.g. [42,43] provide valuable material and should be consulted by those wishing to use non-survey based measures.

Throughout the review, stigma was categorised as perceived, experienced or self stigma. These distinctions were useful for organising the review, however many inter-connections exist between the concepts, and there was sometimes difficulty in judging which was the most appropriate to use in categorising a subscale. This points to the complex nature of stigma, as highlighted in the introduction, and reinforces the necessary interplay of cognitive, affective and behavioural aspects of perceived, experienced and self stigma, in fully understanding the individual's position in relation to stigma.

## Competing interests

The authors declare that they have no competing interests.

## Authors' contributions

EB designed and completed this review as part of her PhD research under the supervision of GT and MS. SC contributed additional material to the background. All authors contributed to revising the manuscript. All authors read and approved the final manuscript.

## Pre-publication history

The pre-publication history for this paper can be accessed here:

http://www.biomedcentral.com/1472-6963/10/80/prepub

## Supplementary Material

Additional file 1 **Description of each study located**. Further information on each of the 57 papers included in this review. Click here for file
