# Supplementary material for: Experiences of mental illness stigma, prejudice and discrimination: a review of measures
Source: BMC Health Serv Res. 2010 Mar 25;10:80. doi: 10.1186/1472-6963-10-80 (PMC2851715; doi:10.1186/1472-6963-10-80)
Supplement: Additional file 1 — Description of each study located. Further information on each of the 57 papers included in this review. [file 1472-6963-10-80-S1.DOC]

| **Study** | **Additional**  **stigma measures used** | **Study population** | **Psychometric properties reported** |
| --- | --- | --- | --- |
| 1. PDD (35) | | | |
| [1] | CESQ, HSS | 83 current or former psychiatric service users England | UK adaptation. 11 items included. Items included if item total correlation >0.40 and internal consistency >0.80 |
| [2] | None | 31 people with a diagnosis of schizophrenia US | None reported |
| [3] | RES | 40 current psychiatric service (outpatient) users with SMI Sweden | Swedish translation  Internal consistency α= 0.87 |
| [4] | None | 182 individuals with SMI US | Psychometrics in [5] reported |
| [6] | CESQ | 193 current psychiatric service (outpatient) users Hong Kong | Chinese translation |
| [7] | None | 14 patients with disassociate disorder and 42 patients with somatoform pain disorder Austria. | German translation |
| [8] | None | 115 patients with somatoform pain disorder | German translation [9] |
| [10] | None | 90 psychiatric patients with schizophrenia or affective disorder | German translation [9] |
| [11] | None | 280 psychiatric inpatients Switzerland | German translation [9] |
| [12] | RES | 92 psychiatric service users Sweden | Swedish translation  Internal consistency α= 0.90 |
| [13] | None | 461 participants with psychiatric disabilities US | Internal consistency for subscales range α=0.79- α=0.95 |
| [14] | None | 127 outpatients with a diagnosis of schizophrenia Germany | Internal consistency α=0.88 |
| [15] | None | 21 psychiatric service users England | None reported |
| [16] | None | 184 people with SMI US | Internal consistency α=0.74 |
| [17] | SRER | 84 men with dual diagnosis of mental disorder and substance abuse | Internal consistency α=0.82 |
| [18] | None | 70 members of a psychiatric rehabilitation clubhouse programme US | Internal consistency α=0.88, 0.86 and 0.88 at baseline, 6 and 24 months |
| [19] | None | 88 members of a psychiatric rehabilitation clubhouse programme US | Internal consistency α=0.88, 0.86 and 0.88 at baseline, 6 and 24 months |
| [20] | SRER | 84 men with dual diagnosis of mental disorder and substance abuse | Used 15-item version, α=0.78 |
| [21] | RES | 200 current or previous psychiatric service users Sweden | As in [3] |
| [22] | RES | 150 current psychiatric service users Sweden | As in [3] |
| [23] | None | 20 psychiatric (inpatient) service users England | None reported |
| [24] | None | 610 members of psychiatric self-help groups or receiving outpatient treatment US | Internal consistency α=0.78 |
| [25] | None | 610 members of psychiatric self-help groups or receiving outpatient treatment US | Internal consistency α=0.78 |
| [26] | None | 165 people with SMI Switzerland | German translation [9] |
| [27] | None | 264 people using psychiatric inpatient or outpatient services US | Internal consistency α = 0.83 |
| [28] | ISMI | 127 psychiatric outpatients US | Internal consistency α = 0.84 |
| [29] | ISMI | 82 psychiatric outpatients US | Internal consistency α = 0.85 |
| [30] | None | 1,187 patients with depression using primary care services US | Adapted version |
| [31] | None | 144 members of a clubhouse programme US | Psychometric properties in [5,32] reported |
| [33] | None | 60 women with borderline personality disorder and 30 women with social phobia Germany | None reported |
| [34] | SSMIS | 60 women with borderline personality disorder and 30 women with social phobia Germany | German translation [9] |
| [35] | None | 134 outpatients taking antidepressants US | Internal consistency α =0.94 |
| [36] | None | 172 outpatients with schizophrenia Switzerland | Psychometrics in (Link 1991) α =0.86-0.88, reported |
| [37] | SRER | 88 former psychiatric patients US | Adapted version of [5] with yes/no response format. No psychometrics reported |
| [38] | None | 104 people with diagnosis of SMI US | Psychometrics in [5] reference |
| 2. ISMI (7) | | | |
| [39] | None | 51 people with schizophrenia spectrum disorders US | Alienation, stereotype endorsement and discrimination experience subscales were used. Psychometrics in [28,29] reported |
| [40] | None | 36 people with schizophrenia US | Psychometrics in [28] reported |
| [41] | None | 75 people with schizophrenia spectrum disorders US | Psychometrics in [28,29] reported |
| [42] | None | 113 adults with diagnosis of schizophrenia or schizoaffective disorder US | Psychometrics in [28] reported |
| [28] | PDD | 127 psychiatric (outpatient) service users US | See Table 2 |
| [29] | PDD | 82 psychiatric (outpatient) service users US | Psychometrics in [28] reported |
| [43] | None | 86 psychiatric (inpatient) service users with schizophrenia Israel | Hebrew version α=0.86.  Internal consistency for subscales range α=0.26 to α=0.70 |
| [44] | None | 102 people with schizophrenia spectrum disorders US | Used only alienation and stereotype endorsement subscales of ISMI |
| 3. SSMIS (5) | | | |
| [45] | None | Study 1, 54 people with psychiatric disabilities. Study 2, 60 people with psychiatric disabilities US | See Table 2 |
| [46] | None | 108 people with SMI in Hong Kong | Chinese translation. Internal consistency ranged from α=0.82-0.90 for the 4 subscales. Test-retest reliability ranged from 0.71-0.81 for the subscales. Factor structure was confirmed |
| [47] | None | 86 people with schizophrenia Hong Kong | Chinese translation. Psychometrics in [46] reported |
| [48] | PDD | 60 women with borderline personality disorder and 30 women with social phobia Germany | German translation. Internal consistency for subscales range α=0.82 to α=0.92. Construct validity examined with empowerment measure |
| [49] | None | 108 people with psychosis Hong Kong | Psychometrics in [45] reported |
| [50] | None | 71 psychiatric (outpatient) service users US | Psychometrics in [45] reported |
| 4. CESQ (3) | | | |
| [1] | None | 83 current or former psychiatric service users England | UK adaptation. 7 items included. Items included if item total correlation >0.40 and internal consistency >0.80 |
| [51] | None | 100 current psychiatric service users US | No properties reported |
| [52] | None | 74 psychiatric (outpatient) service users with schizophrenia US | Modified version of CESQ. No properties reported |
| [53] | None | 1,301 members of NAMI, a mental illness charity US | See Table 2 |
| 5. RES (3) | | | |
| [3] | PDD | 40 current psychiatric service (outpatient) users with SMI Sweden | See Table 2 |
| [12] | None | 92 psychiatric service users Sweden | Internal consistency α=0.83 |
| [21] | PDD | 200 current or previous psychiatric service users Sweden | Psychometrics in [3] reported |
| [22] | PDD | 150 current psychiatric service users Sweden | Psychometrics in [3] reported |
| 6. DSSS (1) | | | |
| [54] | None | 92 African Americans with self-reported low mood US | See Table 2 |
| [55] | None | 168 undergraduates and 223 community members. Depression was measured. | Internal consistency for subscales range α=0.79- α=0.93. Total α=0.95. Factor analysis used to establish scale structure. Construct validity established |
| 7. SRER (1) | | | |
| [20] | PDD | 84 men with dual diagnosis of mental disorder and substance abuse | See Table 2 |
| [37] | PDD | 88 former psychiatric patients US | Used 7 items from scale. No psychometrics reported |
| 8. SS (0) | | | |
| [56] | None | 193 psychiatric service users England. | See Table 2 |
| 9. ISE (0) | | | |
| [57] | None | 88 participants with SMI Canada | See Table 2 |
| 10. SESQ (0) | | | |
| [58] |  | 186 members of a self-help group for manic depression England | See Table 2 |
| 11. HSS (0) | | | |
| [1] | CESQ, HSS | 83 current or former psychiatric service users England | See Table 2 |
| 12. MIDUS (0) | | | |
| [59] | None | National population survey n= 3,032 US. Major depression, general distress and generalized anxiety disorder were measured. | See Table 2 |
| 13. DISC (0) | | | |
| [60] | None | 732 people with schizophrenia in 27 countries | See Table 2 |
| 14. EDS (0) | | | |
| [61] | None | 1,827 people receiving treatment from traditional mental health services US | See Table 2 |

Reference List

1. Bagley C, King M: **Exploration of three stigma scales in 83 users of mental health services: implication for campaigns to reduce stigma.** *Journal of Mental Health* 2005, **14:** 343-355.

2. Berge M, Ranney M: **Self-esteem and stigma among persons with schizophrenia: implications for mental health.** *Care Management Journals* 2005, **6:** 139-144.

3. Bjorkman T, Svensson B, Lundberg B: **Experiences of stigma among people with severe mental illness. Reliability, acceptability and construct validity of the Swedish versions of two stigma scales measuring devaluation/discrimination and rejection experiences.** *Nordic Journal of Psychiatry* 2007, **61:** 332-338.

4. Blankertz L: **Cognitive components of self esteem for individuals with severe mental illness.** *American Journal of Orthopsychiatry* 2001, **71:** 457-465.

5. Link BG: **Understanding labeling effects in the area of mental disorders: an assessment of the effect of expectations of rejection.** *American Journal of Community Psychology* 1987, **11:** 261-273.

6. Chung KF, Wong MC: **Experience of stigma among Chinese mental health patients in Hong Kong.** *Psychiatric Bulletin* 2004, **28:** 451-454.

7. Freidl M, Lang T, Scherer M: **How psychiatric patients perceive the public's stereotype of mental illness.** *Social Psychiatry and Psychiatric Epidemiology* 2003, **38:** 269-275.

8. Freidl M, Spitzl SP, Prause W, Zimprich F, Lehner-Baumgartner E, Baumgartner C *et al*.: **The stigma of mental illness: anticipation and attitudes among patients with epileptic, dissociative or somatoform pain disorder.** *International Review of Psychiatry* 2007, **19:** 123-129.

9. Angermeyer MC. Erfahrungen und Umgang mit psychischer Krankheit. Deutsche fassung der Stigma-Coping-Skalen von Link [German version of Link's Discrimination-Devaluation-Scale]. 1998. Universitätsklinikum, Klinik und Poliklinik für Psychiatrie, Leipzig.

10. Freidl M, Spitzl SP, Aigner M: **How depressive symptoms correlate with stigma perception of mental illness.** *International Review of Psychiatry* 2008, **20:** 510-514.

11. Graf J, Lauber C, Nordt C, Ruesch P, Meyer PC, Rossler W: **Perceived stigmatization of mentally ill people and its consequences for the quality of life in a Swiss population.** *The Journal of Nervous and Mental Disease* 2004, **192**.

12. Hansson L, Bjorkman T: **Empowerment in people with a mental illness: reliability and validity of the Swedish version of an empowerment scale.** *Scandinavian Journal of Caring Sciences* 2005, **19:** 32-38.

13. Kahng SK, Mowbray CT: **What affects self-esteem of persons with psychiatric disabilities: the role of causal attributions of mental illnesses.** *Psychiatric Rehabilitation Journal* 2005, **28:** 354-361.

14. Kleim B, Vauth R, Adam G, Stieglitz RD, Hayward P, Corrigan P: **Perceived stigma predicts low self-efficacy and poor coping in schizophrenia.** *Journal of Mental Health* 2008, **17:** 482-491.

15. Knight MTD, Wykes T, Hayward P: **Group treatment of perceived stigma and self-esteem in schizophrenia: a waiting list trial of efficacy.** *Behavioural and Cognitive Psychotherapy* 2006, **34:** 305-318.

16. Link B, Castille DM, Stuber J: **Stigma and coercion in the context of outpatient treatment for people with mental illnesses.** *Social Science & Medicine* 2008, **67:** 409-419.

17. Link BG, Mirotznik J, Cullen FT: **The effectiveness of stigma coping orientations: can negative consequences of mental illness labeling be avoided?** *Journal of Health and Social Behavior* 1991, **32:** 302-320.

18. Link BG, Struening EL, Neese-Todd S, Asmussen S, Phelan JC: **Stigma as a barrier to recovery: the consequences of stigma for the self-esteem of people with mental illness.** *Psychiatric Services* 2001, **52:** 1621-1626.

19. Link BG, Struening EL, Neese-Todd S, Asmussen S, Phelan JC: **On describing and seeking to change the experience of stigma.** *Psychiatric Rehabilitation Skills* 2002, **6:** 201-231.

20. Link BG, Struening EL, Rahav M, Phelan JC, Nuttbrock L: **On stigma and its consequences: evidence from a longitudinal study of men with dual diagnosis of mental illness and substance abuse.** *Journal of Health and Social Behavior* 1997, **38:** 177-190.

21. Lundberg B, Hansson L, Wentz E, Bjorkman T: **Sociodemographic and clinical factors related to devaluation/discrimination and rejection experiences among users of mental health services.** *Social Psychiatry and Psychiatric Epidemiology* 2007, **42:** 295-300.

22. Lundberg B, Hansson L, Wentz E, Bjorkman T: **Stigma, discrimination, empowerment and social networks: a preliminary investigation of their influence on subjective quality of life in a Swedish sample.** *International Journal of Social Psychiatry* 2008, **54:** 47-55.

23. MacInnes DL, Lewis M: **The evaluation of a short group programme to reduce self-stigma in people with serious and enduring mental health problems.** *Journal of Psychiatric & Mental Health Nursing* 2008, **15:** 59-65.

24. Markowitz FE: **The effects of stigma on the psychological well-being and life satisfaction of persons with mental illness.** *Journal of Health and Social Behavior* 1998, **39:** 335-347.

25. Markowitz FE: **Modeling processes in recovery from mental illness: relationships between symptoms, life satisfaction, and self-concept.** *Journal of Health & Social Behavior 42(1):64-79,* 2001.

26. Mueller B, Nordt C, Lauber C, Rueesch P, Meyer PC, Roessler W: **Social support modifies perceived stigmatization in the first years of mental illness: a longitudinal approach.** *Social Science & Medicine* 2006, **62:** 39-49.

27. Perlick DA, Rosenheck RA, Clarkin JF, Sirey JA, Salahi J, Struening EL *et al*.: **Stigma as a barrier to recovery: adverse effects of perceived stigma on social adaptation of persons diagnosed with bipolar affective disorder.** *Psychiatric Services* 2001, **52:** 1627-1632.

28. Ritsher JB, Otilingam PG, Grajales M: **Internalized stigma of mental illness: psychometric properties of a new measure.** *Psychiatry Research* 2003, **121:** 31-49.

29. Ritsher JB, Phelan JC: **Internalized stigma predicts erosion of morale among psychiatric outpatients.** *Psychiatry Research* 2004, **129:** 257-265.

30. Roeloffs C, Sherbourne C, Unutzer J, Fink A, Tang L, Wells K: **Stigma and depression among primary care patients.** *General Hospital Psychiatry* 2003, **25:** 311-315.

31. Rosenfield S: **Labeling mental illness: the effects of received services and perceived stigma on life satisfaction.** *American Sociological Review* 1997, **62:** 660-672.

32. Link BG, Cullen FT, Struening E, Shrout PE, Dohrenwend BP: **A modified labeling theory approach to mental disorders: an empirical assessment.** *American Sociological Review* 1989, **54:** 400-423.

33. Rusch N, Lieb K, Bohus M, Corrigan P: **Self-stigma, empowerment and perceived legitimacy of discrimination among women with mental illness.** *Psychiatric Services* 2006, **57:** 399-402.

34. Rusch N, Holzer A, Hermann C, Schramm E, Jacob GA, Bohus M *et al*.: **Self-stigma in women with borderline personality disorder and women with social phobia.** *Journal of Nervous and Mental Disease* 2006, **194:** 766-773.

35. Sirey JA, Bruce ML, Alexopoulos GS, Perlick DA, Friedman SJ, Meyers BS: **Stigma as a barrier to recovery: perceived stigma and patient-rated severity of illness as predictors of antidepressant drug adherence.** *Psychiatric Services* 2001, **52:** 1615-1620.

36. Vauth R, Kleim B, Wirtz M, Corrigan P: **Self-efficacy and empowerment as outcomes of self-stigmatizing and coping schizophrenia.** *Psychiatry Research* 2007, **150:** 71-80.

37. Wright ER, Gronfein WP, Owens TJ: **Deinstitutionalization, social rejection, and the self-esteem of former mental patients.** *Journal of Health and Social Behavior* 2000, **41:** 68-90.

38. Yanos PT, Rosenfeld S, Horowitz AV: **Negative and supportive social interactions and quality of life among persons diagnosed with severe mental illness.** *Community Mental Health Journal* 2001, **37:** 405-419.

39. Lysaker PH, Buck KD, Taylor AC, Roe D: **Associations of metacognition and internalized stigma with quantitative assessments of self-experience in narratives of schizophrenia.** *Psychiatry Research* 2008, **157:** 31-38.

40. Lysaker PH, Davis LW, Warman DM, Strasburger A, Beattie N: **Stigma, social function and symptoms in schizophrenia and schizoaffective disorder: associations across 6 months.** *Psychiatry Research* 2007, **149:** 89-95.

41. Lysaker PH, Roe D, Yanos PT: **Toward understanding the insight paradox: internalized stigma moderates the association between insight and social functioning, hope, and self-esteem among people with schizophrenia spectrum disorders.** *Schizophrenia Bulletin* 2007, **33:** 192-199.

42. Lysaker PH, Tsai J, Yanos P, Roe D: **Associations of multiple domains of self-esteem with four dimensions of stigma in schizophrenia.** *Schizophrenia Research* 2008, **98:** 194-200.

43. Werner P, Aviv A, Barak Y: **Self-stigma, self-esteem and age in persons with schizophrenia.** *International Psychogeriatrics* 2007, 1-15.

44. Yanos PT, Roe D, Markus K, Lysaker PH: **Pathways between internalized stigma and outcomes related to recovery in schizophrenia spectrum disorders.** *Psychiatric Services* 2008, **59:** 1437-1442.

45. Corrigan PW, Watson AC, Barr L: **The self-stigma of mental illness: Implications for self-esteem and self-efficacy.** *Journal of Social & Clinical Psychology* 2006, **25:** 875-884.

46. Fung KM, Tsang HW, Corrigan PW, Lam CS, Cheung WM: **Measuring self-stigma of mental illness in China and its implications for recovery.** *International Journal of Social Psychiatry* 2007, **53:** 408-418.

47. Fung KMT, Tsang HWH, Corrigan PW: **Self-stigma of people with schizophrenia as predictor of their adherence to psychosocial treatment.** *Psychiatric Rehabilitation Journal* 2008, **32:** 95-104.

48. Rusch N, Holzer A, Hermann C, Schramm E, Jacob GA, Bohus M *et al*.: **Self-stigma in women with borderline personality disorder and women with social phobia.** *Journal of Nervous & Mental Disease* 2006, **194:** 766-773.

49. Tsang HWH, Fung KMT, Corrigan PW: **Psychosocial treatment compliance scale for people with psychotic disorders.** *Australian and New Zealand Journal of Psychiatry* 2006, **40:** 561-569.

50. Watson AC, Corrigan P, Larson JE, Sells M: **Self-stigma in people with mental illness.** *Schizophrenia Bulletin* 2007, **33:** 1312-1318.

51. Charles H, Manoranjitham SD, Jacob KS: **Stigma and explanatory models among people with schizophrenia and their relatives in Vellore, South India.** *International Journal of Social Psychiatry* 2007, **53:** 325-332.

52. Dickerson FB, Sommerville J, Origoni AE, Ringel NB, Parente F: **Experiences of stigma among outpatients with schizophrenia.** *Schizophrenia Bulletin* 2002, **28:** 143-156.

53. Wahl OF: **Mental health consumers experience of stigma.** *Schizophrenia Bulletin* 1999, **25:** 467-478.

54. Rusch LCM, Kanter JWP, Manos RCM, Weeks CEM: **Depression stigma in a predominantly low income African American sample with elevated depressive symptoms.** *Journal of Nervous & Mental Disease* 2008, **196:** 919-922.

55. Kanter JWP, Rusch LCM, Brondino MJP: **Depression self-stigma: a new measure and preliminary findings.** *Journal of Nervous & Mental Disease* 2008, **196:** 663-670.

56. King M, Dinos S, Shaw J, Watson R, Stevens S, Passetti F *et al*.: **The Stigma Scale: development of a standardised measure of the stigma of mental illness.** *British Journal of Psychiatry* 2007, **190:** 248-254.

57. Stuart H, Milev R, Koller M: **The Inventory of Stigmatizing Experiences: its development and reliability.** *World Psychiatry* 2005, **4:** 33-37.

58. Hayward P, Wong G, Bright JA, Lam D: **Stigma and self-esteem in manic depression: an exploratory study.** *Journal of Affective Disorders* 2002, **69:** 61-67.

59. Kessler RC, Mickelson KD, Williams DR: **The prevalence, distribution, and mental health correlates of perceived discrimination in the United States.** *Journal of Health and Social Behavior* 1999, **40:** 208-230.

60. Thornicroft G, Brohan E, Rose D, Sartorius N, Leese M, for The INDIGO Study Group: **Global pattern of anticipated and experienced discrimination against people with schizophrenia.** *The Lancet* 2009, **373:** 408-415.

61. Thompson VL, Noel JG, Campbell J: **Stigmatization, discrimination, and mental health: the impact of multiple identity status.** *American Journal of Orthopsychiatry* 2004, **74:** 529-544.
